# The balance between two isoforms of LEF-1 regulates colon carcinoma growth

**DOI:** 10.1186/1471-230X-12-53

**Published:** 2012-05-28

**Authors:** Shu-Hong Wang, Ke-Jun Nan, Yao-Chun Wang, Wen-Juan Wang, Tao Tian

**Affiliations:** 1 Department of Medical Oncology, The First Affiliated Hospital of the School of Medicine of Xi'an Jiaotong University, Xi'an, Shaanxi Province, P. R. China; 2 Center for Cell-Biological Therapy and Research, General Hospital of Guangzhou Millitary Command of PLA, Guangzhou, Guangdong Province, P. R. China

## Abstract

**Background:**

Colon cancer is one of the most aggressive human malignancies, with a very poor prognosis. Although it has been suggested that different isoforms of the lymphoid enhancer factor (LEF-1) have opposing biological activities, the biological outcome of aberrant LEF-1 activation in colon cancer is still unclear. The aim of this study was to evaluate the effect of the different LEF-1 phenotypes on the growth of colon carcinoma cell lines. A deeper understanding of these processes might improve the targeted therapies for colon cancer by regulating the expression of LEF-1.

**Methods:**

The role of different isoforms of LEF-1 on the growth of human colon carcinoma cell lines (SW480 and HT-29) was studied using various *in vitro* and *in vivo* assays. *In vitro* proliferation, migration, adhesion and apoptosis of the cells stably transfected of different isoforms of LEF-1 were monitored by MTT assay, carboxyfluorescein diacetate–succinimidyl ester staining, annexin V staining, ECM adhesion assay and transwell assay, respectively. In nude mice, the formation of neovasculature in the tumors formed by our constructed cells was measured by immunohistochemistry. All the data were analyzed using a *t* test, and data were treated as significant when p < 0.05.

**Results:**

Overexpression of truncated LEF-1 (LEF-1-ΔL) in the colon cell lines, SW480 and HT29, inhibited their growth significantly *in vitro* and *in vivo*, but the full-length LEF-1 (LEF-1-FL) promoted the proliferation of HT29. Inactivation of Wnt signaling by LEF-1-ΔL reduced the expression of CXCR4 in colon cell lines, which may lead to a decrease in activities such as migration, adhesion and survival. In nude mice, the formation of neovasculature as well as an increase in tumor volume were inhibited by the short isoform of LEF-1. LEF-1-FL, however, caused an increase in all these parameters compared with controls.

**Conclusions:**

These findings suggest that LEF-1 might play an important role in colon carcinogenesis by acting as a regulator. Enhanced expression of LEF-1-FL, which occurs frequently in colon cancer, may be a new target for clinical therapy.

## Background

Lymphoid enhancer factor 1 (LEF-1) is a member of the high mobility group box family that has important roles in organogenesis and colon cancer progression [1,2]. LEF-1 has no transcriptional activation potential by itself, but it can act as an architectural transcription factor in the assembly of multiprotein enhancer complexes, together with other lymphoid-specific proteins. For example, LEF-1, combined with ALY and other enhancer binding proteins, regulates transcription in the T cell receptor alpha enhancer [3,4]. In addition, LEF-1/TCF proteins have been shown to interact with β-catenin and then transfer it into the cell nucleus for transducing Wnt signals to regulate the downstream genes [5,6].

LEF-1 belongs to the multiple promoter gene family, which is usually aberrantly and differentially active in disease. Multiple promoter genes are often characterized by the furthest 5- promoter producing a full-length polypeptide with activities that differ significantly from those encoded by a second downstream promoter that is located inside the gene [7]. The full-length LEF-1 (LEF-1-FL) isoform, containing the β-catenin binding domain, can promote cell proliferation and inhibit differentiation through Wnt signaling [8]. This LEF-1 function may be abolished by the expression of a truncated LEF-1 protein that lacks the β-catenin binding domain. This shorter LEF-1 transcript (LEF-1-ΔL) is thought to be an inhibitory isoform [9,10], owing to its ability to interact with cofactors that also bind LEF-1-FL, as well as compete for DNA binding sites, and thus has been termed “growth suppressing”.

Both LEF-1 isoforms, however, can stimulate cell differentiation and regulate cellular bio-functions [11,12]. The balance between the full and truncated forms may play an important role in cell development. Interestingly, in many colon cancer cell lines, only the LEF-1-FL protein is expressed strongly, and the expression of the short isoform is inhibited significantly [9,13]. Therefore, it is essential to understand the molecular functions of these two LEF-1 isoforms and identify whether LEF-1 could be a new therapeutic target in colon cancer.

To determine whether the LEF-1 gene is involved in the development of colon cancers, we searched the expression state of the LEF-1 gene in colon carcinomas and cell lines. Full-length LEF-1 is almost always expressed in colon cancers *in situ*, and is displaced gradually by the short isoform in the tissues farther from the carcinomas. To further explore the role of LEF-1 in colon carcinogenesis, we expressed the LEF-1 genes stably into the colon cell lines SW480 and HT29. We demonstrated that overexpression of LEF-1-ΔL significantly inhibited the growth of SW480 and HT29 cells and increased their apoptosis *in vitro*. *In vivo*, the colon cells expressing LEF-1-ΔL slowed the growth of colon tumors, and neo-angiogenesis was decreased notably. In brief, we present evidence that LEF-1 might play an important role in colon carcinogenesis by acting as a regulator. Overexpression of LEF-1-ΔL might be a promising candidate for treatment of colon cancer by sensitizing it to chemotherapeutic drugs.

## Methods

### Patient samples

Tumor samples and adjacent normal tissues were acquired during surgery from 22 untreated cancer patients. The study was approved by the Ethical Committee of the Medical Faculty of Medicine of Xi'an Jiaotong University. Informed consent was obtained from all subjects. Every patient sample was divided into three parts, namely tumor tissues, adjacent tissues (the distance from tumors was greater than 2 cm but less than 5 cm) and normal tissues. All samples were immediately frozen by liquid nitrogen and stored at -80°C before protein extraction.

### Plasmids

To construct pCDNA3.1-His-LEF-1-ΔL and pCDNA3.1-His-LEF-1-FL, the short and long transcripts of LEF-1 were cloned by PCR from a human lymph node cDNA library. After sequencing, the correct clones were cut from pMD-18 T by HindIII and BamH I, and then inserted into pCDNA3.1/V5-His (Invitrogen), to generate target plasmids. LEF-1 variants and the tag did not fuse together and were expressed independently.

The primers used for cloning were as follows:

LEF-1-ΔL F: 5’- GGAAAGCATCCAGATGGAGGC-3’;

LEF-1-ΔL R: 5’- AATGAGCTTCGTTTTCCACCATG -3’;

LEF-1-FL F: 5’- CACAGCGGAGCGGAGATTACA -3’;

LEF-1-FL R: 5’- AATGAGCTTCGTTTTCCACCATG -3’;

### Cell culture and transfection

The human colon cell lines SW480 and HT29 were cultured in RPMI1640 medium supplemented with 10% fetal bovine serum (FBS) and 2 mML glutamine (GIBCO/BRL). SW480 and HT29 cells were stably transfected using Lipofectamine™ 2000 (Invitrogen Life Technologies, Carlsbad, CA) according to the manufacturer’s protocol. Stable cell lines, including SW480-pCDNA3.1/V5-His, SW480-LEF-ΔL, HT29-pCDNA3.1/V5- His, HT29- LEF-1-ΔL and HT29-LEF-1-FL, were selected in the presence of 800 μg/ml G-418 (MERCK, Germany) for SW480 cells and 600 μg/ml for HT29 cells, and were maintained in RPMI1640 containing 10% FBS and 400 μg/ml of G-418.

### MTT assay

Cells of the co-culture were collected on days 1-5 and were then seeded in 96-well plates (4 × 10^3^ cells per well) with 100 μl of the medium. An equal volume of fresh medium containing 20% MTT (5 mg/ml) was added. Cells were incubated further at 37°C for 4 h, followed by the addition of 150 μl of dimethyl sulfoxide (Sigma) to each well and mixing by shaking at room temperature for 10 min. The absorbance was measured at 490 nm. Each experiment was repeated at least three times, and the data were analyzed with the Student’s *t*-test, with P < 0.05 being considered statistically significant.

### Cell cycle analysis

Cells (1 × 10^6^) were collected and washed with PBS, then fixed by incubating in 75% alcohol for 30 min at room temperature. After washing with cold PBS three times, cell were resuspended in 1 ml of PBS containing 40 μg propidium iodide (PI, Sigma) and 100 μg RNase A (Sigma) and incubated at 37°C for 30 min. Samples were analyzed for DNA contents, using a FACScalibur™ (BD Immunocytometry Systems, San Jose, CA). Each experiment was repeated at least three times.

### Apoptosis

Apoptotic cells were detected using the AnnexinV-FITC Apoptosis Detection KIT I (Pharmingen, San Diego, CA), according to the manufacturer’s instructions.

### Caspase activity assays

Caspase-3 fluorogenic substrate, Ac-DEVD-AFC, came from BD Biosciences (San Jose, CA, USA). Caspase activity in cell lysates was determined according to the manufacturer’s instructions, using an Aminco-Bow-man series-2 spectrofluorometer (440/500-nm excitation/emission), and expressed as a fold increase of caspase-3 over the control.

### Plate colony-forming assay

The plate colony-forming assay process mainly refers to the work of Franken NA et al. [14]. Briefly, cells were plated in 35-mm plates at a density of 500 cells per well in the complete medium. Cells were cultured at 37°C in 5% CO_2_ for 14 days. After fixation using methanol for 10 min, the cells were stained with Giemsa stain for 15 min and colonies (with more than 50 cells) were photographed and counted by Image Pro Plus 6.0 software. Each experiment was repeated at least three times, and data were analyzed with one-way ANOVA analysis and LSD-*t* test.

### Migration assay

Chemotaxis experiments were performed in polycarbonate transwell inserts (5 μm pore diameter, Corning Costar Corp.). Soluble SDF-1 (Peprotech) was added in the lower chamber at a concentration of 100 ng/ml. Cells (2 × 10^5^) were seeded in the upper compartment and were cultured at 37°C for 18 h. Migrated cells in the lower chamber were photographed and counted under a microscope.

### ECM adhesion assay

Wells of a 96-well plate with l0 g/L BSA and 5 mg/L matrigel at 4°C overnight. Some wells were left uncoated as negative controls. The coated plates were incubated at 37°C in a CO_2_ incubator for 45-60 min on the next day. Cells were counted and diluted to 4 × 10^5^/ml and 50 μl of the cell suspension was added in each well. After incubation in a CO_2_ incubator at 37°C for 1 h, non-adherent cells were removed by washing with PBS. The number of adherent cells was counted with the MTT assay.

### Western blotting

Whole-cell extracts were prepared by lysing cells with the RIPA buffer (50 mM Tris–HCl, pH7.9, 150 mM NaCl, 0.5 mM EDTA, and 0.5% NP-40, and 0.1 mM PMSF). Proteins were separated by 12% sodium dodecyl sulfate polyacrylamide gel electrophoresis, and were electroblotted onto polyvinylidene difluoride membranes. Membranes were probed using mouse-anti-human LEF-1 (1 C3.1D10, Chemicon International, Temecula, CA), monoclonal anti-β-actin (AC-74, Sigma) at appropriate dilutions, followed by incubation with horseradish peroxidase-conjugated secondary anti-rabbit or anti-mouse IgG antibody (Sigma). In some experiments, mouse-anti-His tag (R94025, Invitrogen) was used. Blots were developed using an enhanced chemiluminescence system (Roche, Basel, Switzerland).

### Tumor formation

The tumor formation method was based on Hu et al. [15]. In detail, cells (5 × 10^6^) were injected subcutaneously into nude mice. Eighteen days after the initial inoculation, tumor growth was monitored every 3 days by measuring the tumor length (L) and width (W) with a sliding caliper. Tumor size was calculated as L × W^2^ × 0.51. Thirty days after the initial inoculation, tumors were excised and weighed. All animal experiments were approved by and performed in accordance with guidelines from the Animal Experiment Administration Committee of the Medicine of Xi'an Jiaotong University, to comply with international humanitarian standards.

### Histology and immunohistochemistry

Tissues were fixed in 4% paraformaldehyde, embedded in OCT, sectioned in 10 μm thicknesses, and stained with hematoxylin and eosin by standard methods. Immunohistochemistry was performed by standard procedures, with rat antimouse CD31 (1:200 dilution; Chemicon International) or rabbit antimouse HIF1α antibody (1:200 dilution; Chemicon International) as the primary antibody. Secondary antibodies included horseradish peroxidase–conjugated goat antirat IgG or antirabbit IgG (Boster BioTec, Wuhan, China). Samples were developed using a standard DAB reagent and were observed under a microscope. Microvessels were counted by different technicians to evaluate density, based on “hot fields,” which showed the most concentrated vessel regions. For quantification, pictures were captured and then pixels were counted by Image-Pro Plus 6.0 software (MediaCybernetics Inc., Bethesda, MD).

### Statistics

Images were imported into Image Pro Plus 6.0 software, and pixels for each color were analyzed to quantitatively represent positively stained cells. Statistical analysis was performed with the SPSS 12.0 program. Results are expressed as mean ± SD. Comparisons between groups were undertaken using an unpaired Student's *t*-test. P < 0.05 was considered statistically significant.

## Results

### LEF-1 expression in primary tissues and cell lines

**Figure 1:**
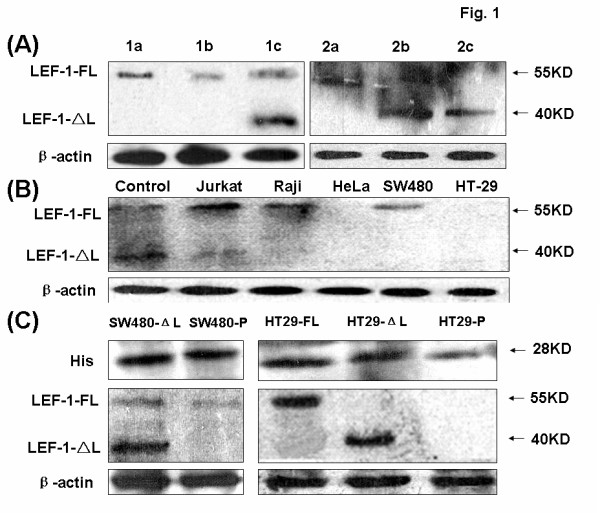
**LEF-1 expression in colon cancer lines and primary tissue.** (**A**) The expression of LEF-1 in primary tissue was examined by western blot. Every sample from the 22 patients was studied, and two representative samples are shown. 1a and 2a denote colon carcinomas; 1b and 2b denote adjacent tissues; 1c and 2c denote normal colon tissues. The molecular weight of full-length LEF-1 is about 55 KD and for LEF-1-ΔL it is 40 KD. (**B**) The expression of LEF-1 in different cell lines was examined by western blot. Control denotes Jurkat cell lysis (Invitrogen). (**C**) The expression of His tag (about 28 KD) and LEF-1 in SW480 and HT29 cells expressing LEF-1-ΔL or LEF-1-FL was examined by western blot. β-Actin was detected as a reference control. SW480-P is short for SW480-pCDNA3.1-His, and HT29-P is short for HT29- pCDNA3.1-His.

To determine whether the expression of LEF-1 is frequent and thus a reliable marker of primary colon carcinoma, we first examined LEF-1 expression in colon tumor tissues and different cell lines. In 81.8% (18/22) of the colon cancer cases we studied, the full-length isoform of LEF-1 was expressed more in colon tumors *in situ* relative to that in adjacent tissues and normal tissues; but the short isoform of LEF-1 was expressed to a lower extent in tumors compared with that in adjacent tissues and normal tissues (Figure 1A). Two forms of LEF-1 could be found in Jurkat and Raji cells, while none were detected in HeLa and HT29 cells (Figure 1B). In another colon cell line, SW480, the balance of LEF-1 expression was broken, since only LEF-1-FL could be detected strongly.

To more thoroughly study the function of LEF-1 in colon cancer, we constructed three colon cell lines that stably expressed the different LEF-1 phenotypes: SW480 expressing truncated LEF-1 (SW480-LEF-1-ΔL), HT29 expressing truncated LEF-1 (HT29- LEF-1- ΔL) and HT29 expressing the full-length LEF-1 (HT29-LEF-1-FL). After selection by G-418, the transfected cells could be detected by the His tag. The patterns of LEF-1 expression in SW480 and HT29 cells indicated that these cells were stably transfected (Figure 1C).

### Truncated LEF-1 could inhibit the growth of the SW480 and HT29 cells

**Figure 2:**
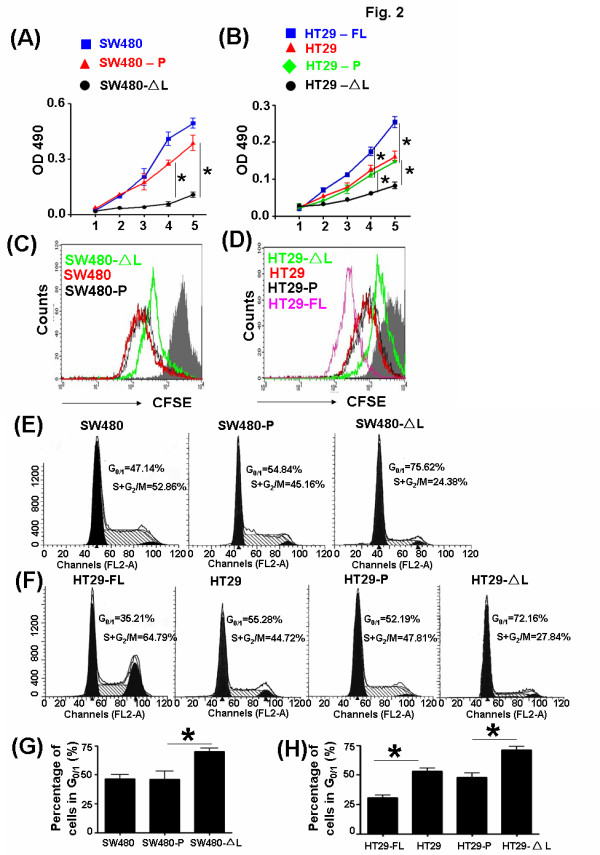
**Proliferation of SW480 and HT29 cells was repressed by LEF-1-**Δ**L.** (**A**) Growth of SW480, SW480-P, and SW480-ΔL cells was compared using the MTT assay. *P < 0.01. (**B**) Growth of HT29, HT29-P, HT29-ΔL, and HT29-FL cells was compared using the MTT assay. *P < 0.05. (**C**) and (**D**) Proliferation of cells in A and B was compared using carboxyfluorescein diacetate–succinimidyl ester staining. (**E**) and (**F**) Cell cycle analysis. Cells in A and B were fixed by 67% ethanol and stained with PI, and cell cycle progression was analyzed by FACS. The numbers in the images are the percentages of cells in G_0/1_ or G_2_ + M stages. Data represent three independent experiments. (**G**) and (**H**) Statistics for the percentages of G0/1 or G2 + M stages in A and B. *P < 0.05.

To investigate the function of LEF-1 on the growth of colon cells, we employed MTT, CSFE staining and cell cycle analysis to detect the proliferation of the stably transfected cells. The growth of SW480 was inhibited by LEF-1-ΔL from day 2 and significant differences in growth could be found on day 5 (Figure 2A). In HT29, LEF-1-ΔL also blocked the proliferation of colon cells, but LEF-1-FL promoted this process, as expected (Figure 2B). To further assess the function of LEF-1 in the proliferation of colon cells, the cells labeled with carboxyfluorescein diacetate–succinimidyl ester were analyzed by FACS after culturing for 3 days. Compared with controls, the proliferation of both SW480 and HT29 that stably expressed LEF-1-ΔL was slower, but HT29 with LEF-1-FL proliferated rapidly (Figure 2C and 2D). We also analyzed the cell cycles of the novel cell lines. LEF-1-ΔL stopped SW480 and HT29 in the G_0/1_ phase (Figure 2E and 2F) and the percentage of cells in G_0/1_ phase increased significantly (Figure 2G and 2H). The full-length form of LEF-1, however, could promote colon cells to begin their cell cycles. These results indicate that LEF-1-FL as the key transcription factor of Wnt signaling, enhanced the proliferation of SW480 and HT29, but LEF-1-ΔL could inhibit the growth of the colon cells.

### Truncated LEF-1 increased apoptosis of SW480 and HT29 cells

**Figure 3:**
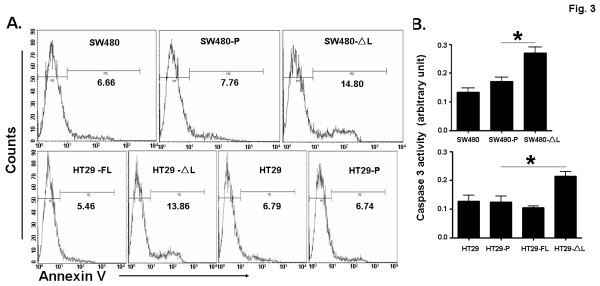
**LEF-1 regulated the survival of colon cell lines.** (**A**) LEF-1-ΔL or LEF-1-FL was expressed stably in SW480 and HT29 cells, and cell death was examined by annexin V-FITC/PI staining. The numbers in images are the percentages of annexin V positive cells. (**B**) The caspase-3 activity was assessed in the cell lines that expressed LEF-1-ΔL or LEF-1-FL. *P < 0.05.

LEF-1 was reported to regulate apoptosis of lymphocytes [16]. We examined the effect of LEF-1 on colon cell apoptosis, using annexin V staining. In SW480 and HT29, the stable expression of LEF-1-ΔL induced more cells to undergo apoptosis, and the percentage of annexin V positive cells was about twice that of controls (Figure 3A). LEF-1-FL reduced the number of apoptotic cells slightly, but no statistical differences could be found (data not shown). Caspase-3 is an executioner caspase, the activation of which represents a distal event in apoptosis signaling pathways. The caspase-3 activity was assessed in both colon cancer cell lines. The caspase-3 activity was increased significantly in the cells that were transfected with LEF-1-ΔL (Figure 3B). These results suggest that truncated LEF-1 interrupts the survival of colon cells.

### LEF-1 regulated the ability of colony formation and adhesion of SW480 and HT29 *in vitro*

**Figure 4:**
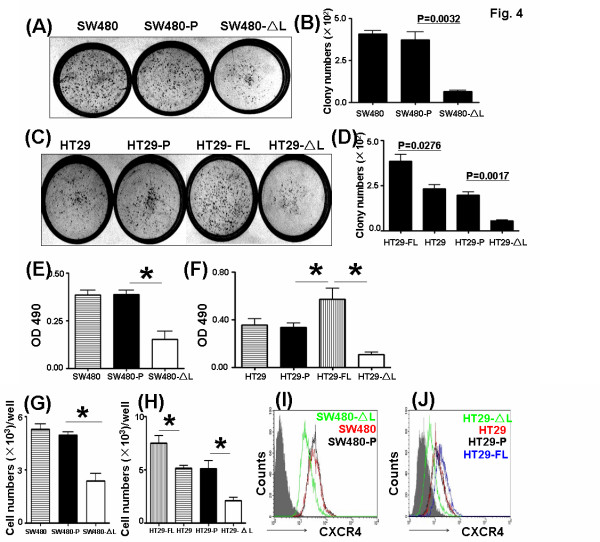
**LEF-1-**Δ**L reduced the abilities of colony formation and adhesion in SW480 and HT29 cells.** (**A**) and (**C**) Colony-forming assay. Fifty SW480 and HT29 cells expressing LEF-1-ΔL or LEF-1-FL were cultured in 35-mm plates, and colonies with more than 50 cells were counted on day 14 after the start of the culture. (**B**) and (**D**) Colonies in A and C were counted under light microscopy. Data are expressed as mean ± S.D. *P < 0.01. (**E**) and (**F**) 2 × 10^4^ cells in A and C were added to wells in a 96-well plate, which was coated with BSA and matrigel. After incubation in a CO_2_ incubator at 37°C for 1 h, non-adherent cells were removed by washing with PBS. The number of adherent cells was counted with the MTT assay. (**G**) and (**H**) Cell migration assay. SW480 and HT29 cells expressing LEF-1-ΔL or LEF-1-FL were seeded in the upper chamber of a trans-well culture system, with SDF1a (100 ng/ml) in the lower chamber. Colon cells migrating into the lower chamber were analyzed by FACS and cell counting. (**I**) and (**J**) The expression of CXCR4 in cells in A and B was analyzed by FACS.

Equal numbers of cells were inoculated in 35-mm plates, and colonies with more than 50 cells were compared after culturing for 14 days. As shown in Figure 4A and 4B, the colony number of SW480-LEF-1-ΔL was significantly lower than that of the control cells, while the colony number of HT29- LEF-1-FL was significantly higher than that of the controls (Figure 4C and 4D). These results indicate that LEF-1 might play an important role in regulating the colony formation ability of the human colon cell lines, SW480 and HT29, *in vitro*. We also examined the ability of these cells to adhere to the ECM. The adherent cells were detected by the MTT assay. SW480 cells expressing LEF-1-ΔL had defects in their ability to adhere, while LEF-1-FL promoted HT29 to adhere to the plates (Figure 4E and 4F). These results indicate that LEF-1 participated in regulating the adhesion of colon cells.

### Truncated LEF-1 reduced the migration of SW480 and HT29 cells

We seeded the same number of cells in the upper compartment of transwell inserts, and SDF-1 was added as a chemotactic factor to the lower chamber. After culturing for 18 h, only a few of the colon cells expressing LEF-1-ΔL arrived at the bottom (Figure 4G and 4H), but significant numbers of HT29 cells with LEF-1-FL passed through the membrane. To clarify the defects in migration, we further investigated the expression of CXCR4, the receptor for SDF-1 on colon cells. The level of CXCR4 was down-regulated by LEF-1-ΔL but up-regulated by LEF-1-FL in the colon cells (Figure 4I and 4J). These results suggest that LEF-1 could regulate the migration of colon cells through CXCR4.

### LEF-1-ΔL inhibited the growth of colon carcinomas in nude mice

**Figure 5:**
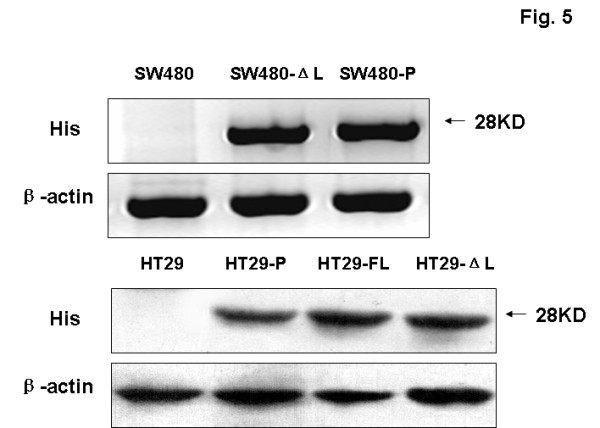
**Expression of the His tag in the tumor cells.** Cell lysates were prepared from different SW480 and HT29 sub-tumors. Western blotting analyses were performed to detect the protein levels of His tag, with β-actin as a reference control.

**Figure 6:**
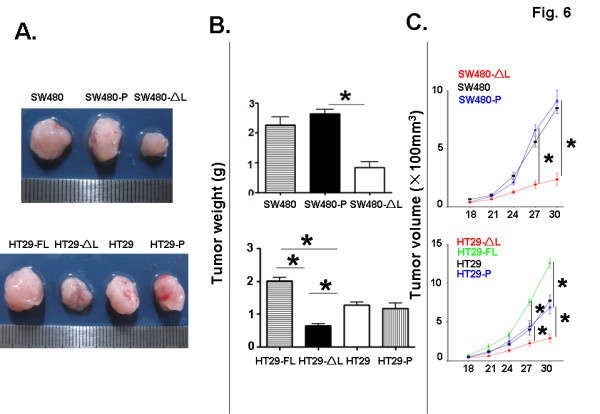
**Tumor formation* in vivo.*** (**A**) Different SW480 and HT29 cell lines were injected subcutaneously into nude mice. Tumors were dissected 30 days after the inoculation and were photographed; representative tumors are shown. (**B**) Tumor weights were compared on day 30 after the tumor inoculation. (**C**) Tumor volume. Tumor volume was monitored every 3 days from day 18 after the inoculation by measuring tumor length (L) and width (W) with a sliding caliper. Tumor volume = L × W^2^ × 0.51. Bars, mean ± SD. *P < 0.05, n > 3.

We further examined the growth of colon cells *in vivo*. We subcutaneously inoculated the modified colon cell lines into nude mice. A His tag could be found in the lysates of tumors, which confirmed the expression of our trans-genes (Figure 5). On day 30 after the inoculation of 5 × 10^6^ cells, the tumors of SW480 and HT29 cells expressing LEF-1-ΔL were significantly smaller than that in mice inoculated with the control cells. Tumors produced by HT29 cells expressing LEF-1-FL were much bigger that that produced by the wild-type HT29 cells (Figure 6A). The tumor weight also indicated that LEF-1-ΔL inhibited the growth of colon carcinomas (Figure 6B). We monitored the rate of tumor volumes from day 18 onward. The volume of tumors produced by SW480 and HT29 cells expressing LEF-1-ΔL was significantly smaller than those in the control mice, and the differences remained even when the tumors grew larger (Figure 6C). These results indicate that LEF-1 could regulate the growth of colon tumors *in vivo.*

### Defective angiogenesis in tumors of colon cells expressing LEF-1-ΔL

**Figure 7:**
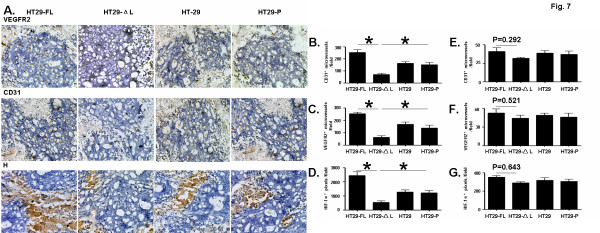
**Density of microvessels and hypoxia in colon tumor tissues.** (**A**) HT29 cells expressing LEF-1-ΔL or LEF-1-FL were injected subcutaneously into nude mice. Thirty days later, the same size tumors were cut into small pieces with approximately equal sizes (about 100 mm^3^) at random, were sectioned in thicknesses of 10 μm and stained for CD31, VEGFR2 and HIF-1α by immunochemistry. (**B**) and (**C**) Microvessels in A labeled with anti-CD31 and anti-VEGFR2 antibodies were counted under a microscope, and microvessel densities (microvessels per field) were compared. (**D**) HIF-1α-positive signals in A were quantified using Image-Pro Puls 5.1 and were compared for statistical significance. (**E**), (**F**) and (**G**) Eighteen days after the initial inoculation of HT29 cells expressing LEF-1-ΔL or LEF-1-FL into nude mice, tumors of different groups were sectioned in thicknesses of 10 μm and stained for CD31, VEGFR2 and HIF-1α by immunochemistry. Positive signals were quantified and compared for statistical significance. Bars, mean ± SD. *P < 0.05, ** P < 0.01; n > 3.

Abortive formation of neovasculature and consequent poor perfusion in solid tumors are considered reasons for tumor regression [17,18]. We therefore examined microvessels in colon tumors produced in nude mice. Immunohistochemical staining of the vasculature with anti-VEGFR2 and anti-CD31 antibodies showed that the microvessel density of tumors formed by HT29-LEF-1-ΔL was decreased significantly compared with the controls (Figure 7A, upper and middle lines). Quantification of microvessels also indicated that the amount of microvessels in tumors formed by HT29-LEF-1-ΔL was lower than those in control tumors (Figure 7B and 7C). We next evaluated tissue hypoxia in tumors by measuring HIF1α expression, using immunohistochemistry. The expression of HIF1α was down-regulated in tumors formed by the colon cells expressing LEF-1-ΔL (Figure 7A, lower line). Quantification analysis also indicated that the hypoxia areas of tumors formed by HT29-LEF-1-ΔL were significantly smaller than those in tumors formed by control cells (Figure 7D). To evaluate whether the reduced tumor vascularization observed in HT29-LEF-1-ΔL also occurred in tumors of the same size, we detected microvessels by immunohistochemistry for CD31, VEGFR2 and HIF1α in 18th-day tumors of different groups that were approximately the same size (about 50 mm^3^; shown in Figure 6C). Tumor vascularization of HT29-LEF-1-ΔL was reduced slightly compared with the controls (shown in Figures 7E7G and 7F), but no statistical differences were observed. These data indicate that in tumors formed by HT29-LEF-1-ΔL, defective vascular network formation might lead to poor perfusion and tissue hypoxia.

## Discussion

LEF-1 is one of the DNA-binding transcription factors that functions in the Wnt signaling pathway, and it acts by recruiting β-catenin to Wnt target genes for regulation. The first exon of the LEF-1 gene encodes the domain that is necessary and sufficient to bind to β-catenin, and a second promoter for transcription is located downstream of this exon. The intronic promoter produces a shorter isoform without the β-catenin binding domain, but retains the DNA binding domain^8^. LEF-1 interacts with transcription co-repressors through a domain in the central portion of the protein. Therefore, shorter polypeptides should function as constitutive transcription repressors or competitive inhibitors of Wnt signaling by binding to Wnt response elements in target genes, thereby disallowing β-catenin access and constitutively inhibiting transcription by recruiting a repressor. Since Wnt signaling directs many important processes during development, and constitutive Wnt signaling is tightly linked to the formation of cancers, the relative expression patterns of activating and repressing LEF isoforms could be important for the Wnt signal throughput to target genes in both normal and abnormal settings.

To understand LEF-1 function in colon cancer, we employed two colon cell lines, SW480 and HT29, to construct stable cell lines expressing each of two LEF-1 isoforms. The full-length form of LEF-1, TCF1 and TCF4 can be detected in the SW480 cell line. But both forms of LEF-1 could not be found in the HT29 cell line, and the only TCF family member expressed in HT29 was TCF4. Okamura et al. [19] and Reya et al. [16] reported that LEF-1 was involved in the regulation of T cell proliferation and apoptosis in pro-B cells. Our data also showed that modulation of LEF-1 resulted in significant changes in colon cell proliferation. As we had hypothesized, LEF-1-ΔL significantly inhibited the proliferation of SW480 and HT29 *in vitro* by blocking the cell cycles at the G_0/1_ phase. Consistent with this, the expression of cyclin D1 and c-Myc, two down-stream target genes of Wnt signaling that can induce S phase entry in many cancer cell lines [20,21], was also decreased under the regulation of the short form of LEF-1 (data not shown). Overexpression of LEF-1-ΔL also induced up-regulation of the Rb protein and down-regulation of cyclin E and D3 (data not shown). This is unusual, because it has been generally accepted that the activity of Rb protein is regulated by phosphorylation catalyzed by cyclin-dependent kinase complexes. Up-regulation of the Rb protein level might neutralize the effect of increased cyclin E and D3 in cell cycle progression, which led to the inhibition of cell proliferation in colon cells.

To our surprise, no increments in the sub G0/G1 population were detected for both SW480-ΔL and HT29-ΔL cells. This would be expected based on the results shown in Figure 3 where an increase in annexin-V positive cells was observed for both these cell lines. We think that there are two main reasons, as follows: (1) As is known, annexin-V, belonging to the protein family of annexins, with anticoagulant properties, has been shown to be a useful tool in detecting early apoptotic cells since it preferentially binds to negatively charged phospholipids such as PS in the presence of Ca2^+^ and shows minimal binding to phosphatidylcholine and sphingomyelin. Changes in PS asymmetry, which are analyzed by measuring annexin-V binding to the cell membrane, were detected before morphological changes associated with apoptosis occurred and before membrane integrity was lost. Apoptotic cells become annexin-V positive after nuclear condensation starts, but before the cell becomes permeable to PI. Therefore, as tools of detection for apoptosis, annexin-V staining is more sensitive than DNA flow cytometric analysis. In our results, the cells we detected may be in a stage of early apoptosis, which could be detected by annexin-V staining but not by cell cycle analysis. (2) SW480 and HT29 cells are adherent and should be disaggregated to have a single cell suspension before annexin-V staining. Any procedure that affects the integrity of the plasma membrane will result in cell positivity for annexin-V. The binding of annexin-V to phosphatidylserine may be affected in adherent cells, which are usually detached from plastic dishes by enzymatic treatment, with their membrane integrity unaltered. Therefore, the apoptosis-positive rate of HT29-dL cells detected by annexin-V staining would be higher than that detected by cell cycle analysis.

We further found that LEF-1 could influence the expression of CXCR4 in SW480 and HT29. It has been demonstrated that SDF-1/CXCR4 signaling, one of the most important chemokine receptor–ligand complexes, was considered to play multiple roles in cell migration, proliferation, chemotactic responses, adhesion, secretion of MMPs and angiopoietic factors in the development of many systems and tumor cells through some possible pathways [22-26]. Luo et al. [27] and others [28-31] have reported that SDF-1/CXCR4 signaling might interact with the Wnt/β-catenin/LEF-1 pathway to regulate the development of the central nervous system. Wang et al. [32] showed that the abrogation of CXCR4 could influence the pancreatic cancer cell phenotype, including cell proliferation, colony formation and cell invasion. Wnt target genes were also inhibited in CXCR4-knockdown cells. Samara et al. [33] found that the binding of CXCR4 to CXCL12 lead to an increase in head and neck squamous cell carcinoma (HNSCC) cell adhesion and MMP-9 secretion, suggesting that CXCR4 may be a novel regulator of metastatic processes in HNSCC. Interestingly, our results indicated that inactivation of Wnt signaling via overexpression of the short form of LEF-1 also down-regulated the expression of CXCR4 in colon cancer cell lines, but CXCR4 expression was enhanced by the full form of LEF-1 in HT29. The changes of CXCR4 in colon cell lines might trigger a series of alterations in cell behavior and biological functions, such as migration, adhesion and microvessel formation. According to our data, the regulation of the CXCR4 protein expression might be a new mechanism in regulating colon cell function by the Wnt pathway.

We further analyzed whether reduced tumor vascularization observed in cells expressing the LEF-1-ΔL variant also occurred in tumors of the same size. As shown in Figure 7, tumor vascularization of HT29-LEF-1-ΔL was reduced slightly compared with the controls, but no statistical differences were obtained. According to our results, colon cell lines expressing LEF-1-ΔL grow much slower than those expressing the full-length both *in vitro* and *in vivo*. As is known, rapid proliferation of tumor cells can cause hypoxia of the internal tumor tissues, which can induce the formation of new blood vessels through high level cytokines such as VEGF. Therefore, the significant difference in microvessels between colon tumors formed by different LEF-1 cell lines may be a result of the difference in the ability for cell proliferation. In the early stage of tumor formation, hypoxia caused by both tumor cells was at a low level, which could not induce statistical differences in the observed microvessels. Therefore, the reduction in tumor vascularization of HT29-LEF-1-ΔL formed gradually during the process of tumor growth, rather than in the initial stages of tumor formation. Though there are many details of tumor formation in colon cell lines with LEF-1 variants to be further understood, the proliferating capability of these cells may be one of the most important inherent initiating agents that can influence tumor growth.

## Conclusions

In summary, our results demonstrated that the balance between the two forms of LEF-1 might have important consequences for normal growth of colon cells and cancer. LEF-1, acting as a central transcription mediator of Wnt signaling and regulator of cell cycle- and growth-relevant genes, can be thought of as a target for clinical treatment and may provide a new direction for the therapy of colon cancer.

## Abbreviations

LEF-1, Lymphoid enhancer factor 1; TCF, T-cell factor; HIF, Hypoxia-inducible factor; VEGF, Vascular endothelial growth factor; CFSE, Carboxyfluorescein diacetate succinimidyl ester; SDF-1, Stromal cell-derived factor-1; CXCR4, C-X-C chemokine receptor type 4; MMP, Matrix metalloproteinase; CDK, Cyclin-dependent kinases.

## Misc

Shu-Hong Wang and Yao-Chun Wang are contributed equally to this work.

## Competing interests

The author(s) declare that they have no competing interests.

## Authors’ contributions

S-H W. and Y-C W. performed most of the experiments including cell culture and prepared the manuscript, examination of cell bio-function and FACS analyses. W-J W. and T T. helped in the construction of plasmids. K-J N. supervised the study. All authors read and approved the final manuscript.

## Pre-publication history

The pre-publication history for this paper can be accessed here:

http://www.biomedcentral.com/1471-230X/12/53/prepub
